# Klotho as an Early Marker of Acute Kidney Injury Following Cardiac Surgery: A Systematic Review

**DOI:** 10.3390/jcdd11050135

**Published:** 2024-04-28

**Authors:** Konstantinos S. Mylonas, Panagiotis Karakitsos, Alireza Tajik, Deanna Pagliuso, Hamidreza Emadzadeh, Ioanna Soukouli, Pouya Hemmati, Dimitrios V. Avgerinos, George T. Stavridis, John N. Boletis

**Affiliations:** 1 Department of Cardiac Surgery, Onassis Cardiac Surgery Center, 17674 Athens, Greece; avgerinos@ocsc.gr (D.V.A.); gstavridis@ocsc.gr (G.T.S.); 2 Department of Cardiology, King’s College Hospital, London SE5 9RS, UK; pakarakitsos@gmail.com; 3 School of Medicine, St. George’s University, True Blue, Grenada; alireza.tajik@uhn.ca (A.T.); hemadzad@sgu.edu (H.E.); 4 York University, Toronto, ON M3J 1P3, Canada; deeanna@my.yorku.ca; 5 Department of Nephrology and Renal Transplantation, Laiko General Hospital, National and Kapodistrian University of Athens, 11527 Athens, Greece; ioanna.s.soukouli@gmail.com (I.S.); inboletis@gmail.com (J.N.B.); 6 Department of Cardiothoracic Surgery, Baptist Health Medical Center, Little Rock, AR 72205, USA

**Keywords:** acute kidney injury, cardiac surgery, Klotho, serum, urine, biomarker, systematic review

## Abstract

Acute kidney injury is a common complication following cardiac surgery (CSA-AKI). Serum creatinine levels require a minimum of 24–48 h to indicate renal injury. Nevertheless, early diagnosis remains critical for improving patient outcomes. A PRISMA-compliant systematic review of the PubMed and CENTRAL databases was performed to assess the role of Klotho as a predictive biomarker for CSA-AKI (end-of-search date: 17 February 2024). An evidence quality assessment of the four included studies was performed with the Newcastle–Ottawa scale. Among the 234 patients studied, 119 (50.8%) developed CSA-AKI postoperatively. Serum Klotho levels above 120 U/L immediately postoperatively correlated with an area under the curve (AUC) of 0.806 and 90% sensitivity. Additionally, a postoperative serum creatinine to Klotho ratio above 0.695 showed 94.7% sensitivity and 87.5% specificity, with an AUC of 92.4%, maintaining its prognostic validity for up to three days. Urinary Klotho immunoreactivity was better maintained in samples obtained via direct catheterization rather than indwelling catheter collection bags. Storage at −80 °C was necessary for delayed testing. Optimal timing for both serum and urine Klotho measurements was from the end of cardiopulmonary bypass to the time of the first ICU lab tests. In conclusion, Klotho could be a promising biomarker for the early diagnosis of CSA-AKI. Standardization of measurement protocols and larger studies are needed to validate these findings.

## 1. Introduction

Acute kidney injury (AKI) develops in approximately 35–40% of patients undergoing cardiac surgery [1]. The underlying processes that lead to postoperative kidney damage remain poorly defined. That said, renal hypoperfusion and non-pulsatile flow during cardiopulmonary bypass (CPB), along with prolonged periods of aortic cross-clamping, CPB, or deep hypothermic circulatory arrest seem to be the primary drivers of morbidity. The development of cardiac surgery-associated AKI (CSA-AKI) is also influenced by microembolization events, hemolysis, and exposure to nephrotoxic medications, particularly in the setting of baseline renal function impairment [2,3,4].

Less than 1% of cardiac surgery patients will need emergency hemodialysis due to severe AKI, and most eventually return to baseline renal function [5]. However, up to 10% of patients will sustain irreversible renal parenchymal damage following cardiac surgery [6]. Perhaps more importantly, even slight increases in serum creatinine levels (0.3–0.5 mg/dL) correlate with inferior 30-day and long-term survival [2]. From a healthcare standpoint, CSA-AKI also prolongs intensive care unit (ICU) and total hospital lengths of stay, thereby increasing aggregate hospitalization expenses and resource utilization [7].

This complex landscape underscores the necessity for early detection and treatment of postoperative renal insufficiency. Although serum creatinine is conventionally utilized as a measure of renal function, its validity is influenced by a variety of factors including muscle mass, dietary intake, and interference from pharmacological agents. Additionally, creatinine concentration may not instantaneously mirror acute tubular injury and its insensitivity may result in undetected renal compromise. The Cleveland Clinic risk score, along with various other predictive models, have attempted to integrate serum creatinine levels and additional variables to enhance prognostic accuracy. Unfortunately, the clinical value of these efforts is debatable [8,9,10]. Research on biomarkers like urinary Insulin-like Growth Factor-Binding Protein-7 (IGFBP-7) and Tissue Inhibitor of Metalloproteinase-2 (TIMP-2) has yielded promising but inconsistent performance across studies, with sensitivity rates ranging from 60 to 100% and specificity rates from 58 to 91% [11]. Neutrophil Gelatinase-Associated Lipocalin (NGAL), cystatin C, Kidney Injury Molecule-1 (KIM-1), and Liver-Type Fatty Acid-Binding Protein (L-FABP) have also yielded equivocal results with regards to their predictive accuracy and reliability [3,12].

On the other hand, the anti-aging hormone Klotho has been long implicated in the pathophysiology of Chronic Kidney Disease (CKD), with seminal studies demonstrating a reduction in its expression with CKD progression [13,14]. Notably, serum Klotho concentrations below 760 pg/mL have been correlated with reduced survival among patients with CKD [15]. Interestingly, Klotho depletion has also been shown to mediate AKI in a variety of conditions including acute myocardial infarction [16], rhabdomyolysis [17], COVID-19 [18], and sepsis [19], as well as platinum [20] and vancomycin toxicity [21]. Although renal histological assessment reveals marked changes at five hours following ischemia-reperfusion injury, a significant drop in renal Klotho protein levels is detectable as early as three hours post-injury [22]. The pathophysiology of this phenomenon seems to be primarily driven by proinflammatory cytokines, oxidative stress, and mitochondrial dysfunction [23,24,25]. This precocious decline in Klotho, antecedent to notable morphological renal alterations, affirms its promising role as an early biomarker for AKI. Despite various investigations into the diagnostic value of Klotho after cardiac surgery, there is a lack of an integrated analysis of these findings [26,27,28]. Our study seeks to bridge this knowledge gap by extensively reviewing the existing literature on the predictive capacity of Klotho for CSA-AKI.

## 2. Relevant Sections

### 2.1. An Overview of Klotho

#### 2.1.1. Klotho History

In 1997, Kuro-o et al. identified a gene whose mutation manifests in a syndrome mimicking advanced aging. The gene was aptly designated “*Klotho*”, drawing inspiration from the mythological Greek figure responsible for spinning the thread of life [29].

#### 2.1.2. Klotho Structure and Function

Klotho predominantly resides in renal distal convoluted tubules, parathyroid glands, and the choroid plexus. Recent findings also show its presence in vascular tissues and the myocardium [13,14,30,31], The gene for Klotho is found on chromosome 13q13.1, and it is responsible for producing a family of enzymes including α-Klotho, which is the most prevalent, as well as β- and γ-Klotho [13].

The soluble form of α-Klotho originates from the cleavage of its membrane-bound precursor by enzymes like ADAM-17. It can also be directly secreted after alternative mRNA splicing [29]. This form can be quantified in urine, serum, and cerebrospinal fluid. Klotho functions as a protective factor against aging via promoting antioxidation, membrane ion transport modulation, inhibition of Wnt signaling pathways, and protection against cellular apoptosis and senescence [32].

α-Klotho is an integral regulator of the FGF-23/1,25-dihydroxyvitamin D/parathyroid hormone axis, essential for maintaining calcium and phosphate equilibrium [33]. It specifically modulates cellular calcium dynamics by upregulating TRPV5 expression and activity, leading to diminished phosphate reabsorption in the kidneys. It also downregulates TRPC6, resulting in reduced intestinal phosphate absorption. Klotho enhances calcium reabsorption in the renal distal tubules by inhibiting endocytosis and by stabilizing the principal calcium channels, particularly TRPV5 and TRPV6 [34,35]. Moreover, its interaction with FGF-23 promotes phosphaturia and mitigates hypercalciuria, preventing mineral supersaturation in the urine [33].

### 2.2. Methods

#### 2.2.1. Search Strategy and Study Selection Process

The current review was performed according to the Preferred Reporting Items for Systematic Reviews and Meta-Analyses (PRISMA) guidelines (end-of search date: 17 February 2024) [36]. Three independent investigators (A.T., D.P., H.E.) conducted a search of the PubΜed/MEDLINE and CENTRAL/Cochrane databases for studies measuring Klotho levels following cardiac surgery procedures. The search criteria were: (cardiac surgery OR valve OR CABG OR aortic aneurysm OR aortic dissection OR heart transplant OR cardiac transplant) AND Klotho. Eligible studies included those that: (1) assessed the relationship between α-Klotho levels and CSA-AKI, (2) used validated assays for α-Klotho in blood, urine, or tissue, and (3) reported CSA-AKI incidence as a primary outcome. Studies lacking α-Klotho level reports in AKI post-cardiac surgery were excluded. All conflicts were resolved following quality control discussions with the first author (KSM). 

#### 2.2.2. Data Extraction

A predefined set of criteria was delineated for the systematic extraction of data from the selected articles. This endeavor was independently conducted by three reviewers (P.K., A.T., D.P.). Any discrepancies were reconciled through the oversight of an additional reviewer (KSM). Extracted data included the methodological framework of each study, demographic profiles, the spectrum of concurrent comorbidities, the incidence and temporality of AKI utilizing established criteria, the nature of the cardiac procedure, the duration of intraoperative events (specifically aortic cross-clamp and CPB durations), Klotho levels gauged at baseline and subsequent postoperative junctures (2, 4, 12, 24, 48, 72 h) whenever possible, the length of ICU and total hospital stay, mortality rates, and the duration of postoperative surveillance. Creatinine values were standardized to mg/dL if alternative units were used in the original studies.

#### 2.2.3. Descriptive Analysis

For continuous data that were reported as median and interquartile (IQR) range, we used the Hozo et al. formula to estimate the respective mean and standard deviation (SD) [37]. When results were not offered in SD, but rather in confidence intervals (CI), we used Cochrane’s handbook equation for converting CI to SD [38]. Continuous data were summarized as means and standard deviations, whereas relative rates were calculated for categorical data. Statistical analysis was performed in RStudio version 2023.03.1 (Boston, MA, USA).

#### 2.2.4. Assessment of Study Quality

The Newcastle–Ottawa Quality scale was used to evaluate the selected studies [39]. A score of at least six indicated high quality. Two reviewers (PK, KSM) independently rated all primary papers and any disagreements were resolved via discussion.

#### 2.2.5. Registration

This systematic review has been registered with the INPLASTY registry and carries the registration number INPLASY202440021 (DOI: 10.37766/inplasy2024.4.0021).

### 2.3. Results

Our initial literature search yielded 98 potentially relevant articles. After reading the titles and abstracts of those, 30 full texts were retrieved for detailed assessment. Ultimately, four studies were included in the present systematic review (Supplemental Figure S1) [26,27,28,40]. A quality assessment of the included studies is shown in Supplemental Table S1. Every study achieved a score of eight, signifying a standard of good quality across all examined research.

In total, 234 cardiac surgery patients were included. Of these, 119 (50.8%) developed AKI postoperatively. The mean age in the AKI group was 64.1 (±13.6) versus 62.1 (±12.2) years in the non-AKI arm. Male was the predominant sex in both subgroups. The design and basic characteristics of the included studies are shown in Table 1. Hypertension was found in 25.9% of the patients in the AKI versus 28% in the non-AKI group and diabetes mellitus in 24% of the renal impairment group versus 18% in the non-AKI arm. Additional co-morbidities, including congestive heart failure, peripheral artery disease, and cerebrovascular disease are summarized in Supplemental Table S2.

Aortic cross-clamp time was longer in the AKI cohort (64.1 ± 32.9 min) compared to the non-AKI cohort (56.1 ± 34.6 min). CPB duration also differed, with the AKI group exhibiting a mean of 101.8 ± 41.7 min, which was substantially longer than the non-AKI cohort (88.8 ± 48.7 min). The length of hospital stay was prolonged in the AKI group, with patients requiring an average of 21.1 days of care, as opposed to 14.1 days for those without AKI. Further details regarding perioperative metrics can be found in Table 2.

The baseline estimated Glomerular Filtration Rate and serum creatinine concentrations were within normal ranges and did not differ significantly between the two cohorts, as shown in Table 3. AKI was detected at the 24 h postoperative interval, with the AKI group presenting a mean serum creatinine level of 1.38 ± 0.52 mg/dL, which was notably higher than the 0.8 ± 0.22 mg/dL observed in patients who did not develop AKI. The divergence in mean serum creatinine levels was more pronounced at the 48 h evaluation, with the AKI group showing 1.49 ± 0.74 mg/dL compared to 0.77 ± 0.23 mg/dL in the non-AKI group. A similar trend, though attenuated, was evident at the 72 h mark.

Serum Klotho levels were specified in two studies at various time points (baseline, immediately postoperatively, as well as at the 2, 4, 24, 48, and 72 h marks) [28,40]. Different units of measurement were employed in the source literature and, therefore, no summative statistics could be computed (Table 4). Similarly, urine Klotho levels were quantified in two distinct studies at varying time points with heterogeneous collection methods precluding the calculation of combined estimates (Table 5) [26,27].

## 3. Discussion

Serum creatinine is traditionally used to assess kidney function in the postoperative setting. Nevertheless, its reliability can be affected by various factors such as race, sex, muscle mass, hydration, diet, and medications [41]. Furthermore, creatinine levels may lag and, occasionally, even fail to convey underlying tubular damage [42,43]. In the context of cardiac surgery, even minimal renal dysfunction can have a profound impact on postoperative outcomes, including patient survival. It is therefore critical to identify and address renal impairment promptly. Research into various biomarkers, including NGAL, has produced equivocal outcomes in terms of their prognostic precision and dependability [3,12]. On the other hand, quantifiable Klotho variations within renal tissue, serum, and urine are consistently observed during the initial hours post-renal injury [22]. This comprehensive review underscores the emerging role of Klotho as an early biomarker for the diagnosis of CSA-AKI.

A total of 234 patients with baseline normal renal function were analyzed. Overall, 50.8% developed CSA-AKI. Criteria for the diagnosis of AKI based on creatinine levels alone were typically met at 24 h postoperatively, and peak values were seen at 48 h after the index operation. The length of hospital stay for patients with AKI was, on average, seven days longer than that of those who did not develop this complication.

Liu et al. measured significantly reduced serum Klotho levels in patients with CSA-AKI at the time of surgery completion (101.97 ± 16.93 U/L) compared to baseline (121.64 ± 19.87 U/L) [40]. Serum Klotho was most effective for early AKI diagnosis immediately postoperatively, achieving an area under the curve (AUC) of 0.806 (95% confidence interval: 0.65 to 0.94, *p* = 0.003) with a diagnostic sensitivity of 89.5% at a threshold of 119.145 U/L [12]. The ratio of serum creatinine to Klotho (SCr/Kl) increased notably in AKI patients in the immediate postoperative period (0.99 ± 0.23 vs. 0.62 ± 0.15, *p* < 0.01) and offered superior AKI detection at a 0.695 cutoff, with 94.7% sensitivity, 87.5% specificity, and 92.4% AUC. Although serum Klotho normalized rapidly within 14 h, the SCr/Kl ratio remained a highly predictive AKI marker for up to three days, with AUC values ranging from 0.82 to 0.90 as well as consistent sensitivity (84.2–94.7%) and specificity (68.7–87.5%) [44].

A subsequent study by a European group reported similar results [28]. Herein, serum Klotho levels dropped to half of their preoperative values when CPB ceased, surged above baseline within 24 h, and ultimately normalized within 48 h. The authors subsequently utilized decision tree analysis, incorporating both Klotho and creatinine levels (measured at the end of surgery, two hours post-surgery, and their changes from preoperative levels) to develop a model with high diagnostic accuracy for CSA-AKI (AUC of 0.92). Overall, these findings suggest an intelligent and simple approach for early detection of AKI by measuring serum Klotho and the SCr/Kl ratio immediately after CPB cessation. Postponing to the point of initial lab testing in the ICU for logistical reasons does not seem to jeopardize the validity of this novel approach.

On the other hand, urinary Klotho is highly unstable, exhibiting rapid reductions in detectable immunoreactivity when stored in human urine [45]. For accurate quantification, stringent handling protocols must be followed, ideally including the prompt analysis of urine samples obtained directly from a catheter. Klotho concentrations seem to decrease by 82% when stored at 37 °C for a duration of three hours either in an indwelling catheter or in the bladder [45]. This reduction can be partly mitigated by the addition of protease inhibitors or 0.1% albumin to freshly collected urine. Nevertheless, the stabilizing effect of these additives is compromised upon freezing. Indeed, an average of an 82% decrease in α-Klotho levels is observed after storage at −20 °C [45]. Conversely, storage at −80 °C preserves at least 60% of α-Klotho concentrations. Subsequent freeze–thaw cycles induce further diminution of α-Klotho levels, irrespective of whether samples are conserved at −80 °C or −20 °C. The mechanisms driving this significant loss may include freeze–thaw cycles, glycosylation loss, structural changes, or decomposition into fragments that are not detected by standard assays [45].

Preliminary clinical research into urinary Klotho post-cardiac surgery documented increased levels in patients with AKI compared to healthy individuals [26]. When contrasting these findings with cardiac surgery patients who did not develop AKI, a statistically significant difference was observed only with the ELISA kit from Shanghai Sunred Biological Technology Co., Ltd. (Shanghai, China), and not with the gold standard kit by IBL International (Human soluble a-Klotho Assay Kit–IBL, Immuno-Biological Laboratories Co., Ltd., Hamburg, Germany) [26]. These conflicting data were constrained by the small sample size of patients with postoperative renal dysfunction and inexperience with handling samples for Klotho-centered ELISA [26]. Indeed, in this study, urine samples were obtained from indwelling catheter collecting bags 12 h postoperatively. Although these samples were subsequently frozen at −80 °C, the concentration and immunoreactivity of α-Klotho level was most likely already compromised by that time.

A more robust patient series recently reassessed and confirmed the value of urinary Klotho in predicting CSA-AKI [27]. In this work, urine samples remained at 4 °C for less than 4 h. After centrifugation at 3000 rpm for five minutes, the supernatants were aliquoted and frozen at −80 °C. Thawing was performed for the first time at the time of Klotho measurement. Baseline renal function and preoperative urinary Klotho levels did not differ between patients with AKI and patients without AKI [27]. The AKIN criteria were implemented to classify renal function decline postoperatively. Creatinine elevation reached the AKI diagnostic threshold 24 h after ICU admission. On the other hand, urinary Klotho demonstrated high predictive accuracy immediately post-surgery with an AUC of 0.86. As a matter of fact, urinary Klotho levels in patients with AKI peaked at the time of transfer to the ICU (1.69 ng/µmol), increasing threefold from baseline measurements. This heightened response persisted for up to three days.

In comparison, urinary NGAL levels peaked significantly later than Klotho, at about two hours post-ICU admission, and returned to baseline within four hours [27]. Within this context, urinary Klotho proved superior to NGAL in predicting AKI both immediately post-surgery and at four hours following admission to the ICU. Patients with AKI stages II and III exhibited a more pronounced rise in urinary Klotho levels compared to those at stage I and individuals without AKI. This trend was consistent from the initial postoperative measurement up to the seventh day [27]. From a pathophysiology standpoint, it seems that the loss of the brush border membrane and the Klotho protein in proximal tubular epithelium, followed by cellular necrosis and exfoliation, leads to increased urinary Klotho [46]. This surge in Klotho is indicative of tubular injury severity and serves as an early indicator of renal cell damage [46].

The Sheba Medical Center in Israel is recognized for conducting the only available pediatric study looking at Klotho following congenital cardiac procedures [47]. In this series, the mean patient age and weight at the time of surgery were 2.16 years and 10.46 kg, respectively. Nearly half of the patients underwent procedures for cyanotic heart disease, including 17.2% for hypoplastic left heart syndrome, 17.2% for tetralogy of Fallot, and 3.5% for D-transposition of the great arteries. Herein, serum Klotho levels significantly declined two hours postoperatively but returned to baseline after six hours. In this study, Klotho levels were not associated with the incidence of AKI. Nevertheless, it should be emphasized that less than 3% of the pediatric cohort developed AKI. Conversely, low Klotho levels at two hours postoperatively (below 785 mg/dL) correlated with a 20-fold increased risk of postoperative complications. This was defined as a composite outcome including not only AKI but also arrhythmias, hemodynamic instability, atelectasis, ventilatory support challenges, and seizures. Furthermore, throughout the four time points measured, Klotho levels were consistently lower in children experiencing postoperative complications. Interestingly, Klotho concentrations above 1068 mg/dL at two hours were associated with a 98.9% lower risk of thrombocytopenia 72 h following CPB. These findings imply a potential protective effect of Klotho in reducing the duration of surgery-induced thrombocytopenia and highlight the need for additional research to explore the relationship between soluble Klotho levels and platelet function.

As previously described, serum Klotho levels seem to normalize within 14 to 48 h following CSA-AKI, possibly as a protective homeostatic mechanism against further renal damage [28,40]. This rebound could provide insights for Klotho’s therapeutic application in AKI management. Of note, exogenous administration of Klotho seems to improve histopathological renal changes and enhance renal function in mice with contrast-induced AKI. This appears to be associated with reduced production of reactive oxygen species (ROS), as well as diminished serum superoxide dismutase and malondialdehyde levels following treatment [48]. In animal models, Klotho repletion also reduced the expression of pro-inflammatory markers p-NF-κB and pyroptosis-related proteins, such as NLRP3, the pro-apoptotic caspase-1, GSDMD, and cleaved GSDMD [48]. Klotho diminishes the cellular surface expression of organic cation transporter 2 and, therefore, suppresses the intracellular uptake of nephrotoxins [20]. KP1, a novel peptide derivative of Klotho, has also been shown to mitigate tubular cell injury and apoptosis induced by SARS-CoV-2 N protein in mice [18]. This protective effect may stem from its capacity to inhibit the TGF-β/Smad signaling pathway. Moreover, repeated doses of recombinant Klotho, at 20 μg/kg every two days, boosted antioxidant defenses in mice via the JAK2/STAT3/GPx3 pathway, thereby mitigating vancomycin-induced AKI [21]. Similarly, administration of recombinant Klotho in septic mice alleviated sepsis-induced AKI by activating Nrf2 to inhibit the ferroptosis signaling pathway [19]. While yet unexplored, similar effects may be observed in the therapeutic use of Klotho for treating CSA-AKI in humans.

Our review has several limitations. First, only four small observational studies were eligible for data analysis. Furthermore, just two studies provided serum Klotho measurements [28,40] and only two quantified urinary Klotho [26,27]. Timing, philosophy of collection, storage, and units of measurements also varied significantly among the cited literature. Moreover, different ELISA kits and calibration methods were utilized across studies, with one group not specifying which commercial kit was employed [40]. In addition, there was significant discrepancy with regards to the criteria that were utilized to define and stage acute kidney injury. Because of these multi-faceted limitations, we could not perform meaningful meta-analysis of Klotho-centered outcomes. Due to scarcity of data, conducting a direct comparison of Klotho to NGAL, cystatin C, or other biomarkers was also not feasible. Lastly, we observed inconsistent reporting of transfusion rates, cardiac index, CPB flow, hemoglobin values and other factors associated with AKI across the source studies. This heterogeneity in data reporting constrained our ability to comprehensively analyze these potentially critical factors in relation to AKI development post-cardiac surgery.

## 4. Conclusions

The present review reinforces the diagnostic value of Klotho as a biomarker for CSA-AKI. The earliest postoperative diagnosis of AKI using creatinine is possible at 24 h. Serum Klotho levels at a threshold of 120 U/L yield an AUC of 0.806 and a diagnostic sensitivity of nearly 90%. The ratio of serum creatinine to Klotho immediately postoperatively with a cutoff of 0.695 shows high predictive power, with 94.7% sensitivity, 87.5% specificity, and an AUC of 92.4%, maintaining its prognostic utility for up to three days. The optimal timing for these measurements is from the cessation of CPB to the first laboratory assessments in the ICU. For urinary Klotho, immediate analysis of samples collected directly from a catheter is recommended. If delayed analysis is required, samples should be stored at −80 °C. Lastly, the therapeutic potential of recombinant Klotho, predicated upon its antioxidative properties, opens new avenues for AKI treatment and warrants further investigation.

## 5. Future Directions

Additional data are needed before Klotho can be utilized in everyday clinical practice. Standardization in measurement protocols, including collection, timing, handling, storage, and unit reporting, is essential to establish diagnostic benchmarks that may enable earlier detection of CSA-AKI than current creatinine-based methods. Furthermore, the implications of Klotho in pediatric cardiac surgery remain to be adequately investigated. Lastly, the therapeutic mechanisms of Klotho warrant deeper exploration through translational and clinical trials. Although we are not there yet, perhaps Klotho might be the key to the diagnostic and treatment conundrum of CSA-AKI.

## Figures and Tables

**Table 1:** Study characteristics.

Study ID	Design	Inclusion Criteria	Exclusion Criteria	Sample	AKI Criteria System	AKI—n (%)	AKI—Mean Age (Years)	Non AKI—Mean Age (Years)	AKI Male (%)	Non AKI Male (%)	Measured Biomarker
Torregrosa et al., 2015 [26]	Observational study	Patients with ACS or heart failure secondary to coronary or valvular disease who underwent either coronary angiography or cardiac surgery	Age younger than 18 years, CKD on replacement therapy, AKI secondary to cardiogenic shock during hospitalization	30	RIFLE	15 (50%)	67	68	13 (86%)	10 (66%)	Postoperative urine Klotho
Yong-Jun Liu et al., 2015 [40]	Observational study	Patients undergoing cardiac valve surgery	Pre-existing renal insufficiency, severe heart failure, perioperative nephrotoxic drug use, postoperative low cardiac output syndrome	35	AKIN	19 (54%)	52.5	51.8	7 (36%)	5 (31%)	Pre- and post- operative serum Klotho
Yingying Qian et al., 2019 [27]	Observational study	Patients undergoing cardiac surgery (CABG, valve, CABG plus valve, congenital heart disease, aortic aneurysm surgery)	Chronic kidney disease, thyroid disease, pre-op usage of high-dose corticosteroids, pre-existing UTI, and missing clinical data	91	AKIN	33 (36%)	64.1	60.4	23 (69%)	35 (60%)	Postoperative urine Klotho
Ales Jerin et al., 2020 [28]	Observational study	Patients undergoing elective cardiac surgery	History of kidney disease including diabetic nephropathy, renal transplantation, malignancy, autoimmune diseases and pregnancy	78	KDIGO	52 (66%)	72.73	68.3	27 (52%)	13 (50%)	Pre- and postoperative serum Klotho
***Total***				*234*		*119 (50.8%)*	*64.1 ± 13.6*	*62.1 ± 12.2*	*70 (58%)*	*63 (54%)*	

ID: identification; n: number of patients; ACS: acute coronary syndrome; AKI: acute kidney injury; CABG: coronary artery bypass graft surgery; CKD: chronic kidney disease; UTI: urinary tract infection; NA: not applicable; NR: not reported.

**Table 2:** Perioperative characteristics.

Study ID	Type of Cardiac Surgery—n (% of Total Study Patients)					Cross Clamp Time (Minutes)		Cardiopulmonary Bypass Time (Minutes)		Length of Hospital Stay (Days)		Length of ICU Stay (Days)		Mortality	
	***CABG***	***Valve***	***CABG + Valve***	***Aortic***	***Congenital (Cyanotic vs. Non-Cyanotic)***	***AKI***	***Non-AKI***	***AKI***	***Non-AKI***	***AKI***	***Non-AKI***	***AKI***	***Non-AKI***	***AKI***	***Non-AKI***
Torregrosa et al., 2014 [26]	NR	NR	NR	NR	NR	NR	NR	NR	NR	27 ± 24	17 ± 10	NR	NR	4/15 (26%)	0/15 (0%)
Yong-Jun Liu et al., 2015 [40]	NR	35 (100%)	NR	NR	NR	70.1 ± 22.2 *	58.5 ± 24.1	100.5 ± 28.1 *	88.2 ± 25.2	NR	NR	NR	NR	NR	NR
Yingying Qian et al., 2019 [27]	32 (35.2%)	39 (42%)	10 (11%)	5 (5.5%)	5 (5.5%)	46.6 ± 45.9	37.5 ± 49.3	102.8 ± 60.2	84.8 ± 88.7	NR	NR	NR	NR	NR	NR
Ales Jerin et al., 2020 [28]	NR	NR	NR	NR	NR	75.5 ± 30.6	72.3 ± 30.4	100.2 ± 36.8	93.6 ± 32.4	15.2 ± 18.5	11.3 ± 7.3	7.9 ± 7.9	5 ± 5	NR	NR
***Total***						*64.1 ± 32.9*	*56.1 ± 34.6*	*101.8 ± 41.7*	*88.8 ± 48.7*	*21.1 ± 21.2*	*14.1 ± 8.6*				

ID: identification; n: number of patients; CABG: coronary artery bypass graft; AKI: acute kidney injury; ICU: intensive care unit; NR: not reported; NA: not applicable; *: *p* < 0.005.

**Table 3:** Serum creatinine.

Study ID	Baseline eGFR (mL/min/1.73 m^2^—as Presented in Each Study)		Baseline Serum Creatinine (mg/dL)		Immediate Post-Op Serum Creatinine (mg/dL)		2-h Post-Op Serum Creatinine (mg/dL)		4-h Post-Op Serum Creatinine (mg/dL)		24-h Post-Op Serum Creatinine (mg/dL)		48-h Post-Op Serum Creatinine (mg/dL)		72-h Post-Op Serum Creatinine (mg/dL)	
	AKI	Non-AKI	AKI	Non-AKI	AKI	Non-AKI	AKI	Non-AKI	AKI	Non-AKI	AKI	Non-AKI	AKI	Non-AKI	AKI	Non-AKI
Torregrosa et al., 2015 [26]	62 ± 29	60 ± 16	1.18 ± 0.51	1.15 ± 0.37	NR	NR	NR	NR	NR	NR	NR	NR	NR	NR	NR	NR
Yong-Jun Liu et al., 2015 [40]	93.82 ± 17.58	96.09 ± 19.65	0.83 ± 0.13	0.83 ± 0.19	1.12 ± 0.19 *	0.84 ± 0.14	NR	NR	1.41 ± 0.24	0.97 ± 0.23	1.45 ± 0.49	0.81 ± 0.23	1.48 ± 0.70	0.81 ± 0.24	1.36 ± 0.71	0.80 ± 0.26
Yingying Qian et al., 2019 [27]	88.38 ± 14.35	84.27 ± 17.37	0.86 ± 0.17	0.81 ± 0.14	NR	NR	NR	NR	NR	NR	NR	NR	NR	NR	NR	NR
Ales Jerin et al., 2020 [28]	117.31 ± 54.71	102.7 ± 26.2	0.86 ± 0.44	0.75 ± 0.17	0.92 ± 0.33	0.73 ± 0.18	1.02 ± 0.38	0.81 ± 0.14	NR	NR	1.32 ± 0.56	0.79 ± 0.22	1.51 ± 0.79	0.74 ± 0.23	NR	NR
***Total***	*90.37 ± 28.91*	*85.76 ± 19.80*	*0.93 ± 0.31*	*0.88 ± 0.22*	1.02 ± 0.26 *	*0.78 ± 0.16*	*1.02 ± 0.38*	*0.81 ± 0.14*	*1.41 ± 0.24*	*0.97 ± 0.23*	*1.38 ± 0.52*	*0.8 ± 0.22*	*1.49 ± 0.74*	*0.77 ± 0.23*	*1.36 ± 0.71*	*0.80 ± 0.26*

* *p* < 0.05; AKI: acute kidney injury; NR: not reported.

**Table 4:** Serum Klotho.

Study ID	Baseline Serum Klotho		Immediate Post-Op Serum Klotho		2-Hour Post-Op Serum Klotho		4-h Post-Op Serum Klotho		24-h Post-Op Serum Klotho		48-h Post-Op Serum Klotho		72-h Post-Op Serum Klotho	
	***AKI***	***Non-AKI***	***AKI***	***Non-AKI***	***AKI***	***Non-AKI***	***AKI***	***Non-AKI***	***AKI***	***Non-AKI***	***AKI***	***Non-AKI***	***AKI***	***Non-AKI***
Torregrosa et al., 2015 [26]	NR	NR	NR	NR	NR	NR	NR	NR	NR	NR	NR	NR	NR	NR
Yong-Jun Liu et al., 2015 [40]	121.64 ± 19.87 ^1^	122.76 ± 20.18	101.97 ± 16.93 *	124.40 ± 20.66	NR	NR	102.77 ± 14.44	118.1 ± 18.74	111.85 ± 11.78	120.43 ± 17.55	116.58 ± 12.73	126.29 ± 17.78	120.50 ± 13.17	128.67 ± 18.84
Yingying Qian et al., 2019 [27]	NR	NR	NR	NR	NR	NR	NR	NR	NR	NR	NR	NR	NR	NR
Ales Jerin et al., 2020 [28]	1.82 ± 0.99 ^2^	1.75 ± 1.03	0.97 ± 0.86	0.70 ± 0.72	2.20 ± 1.27	1.83 ± 0.95	NR	NR	1.73 ± 0.78 ^+^	1.60 ± 0.92	1.63 ± 0.77	1.40 ± 0.78	NR	NR
Total	NA	NA	NA	NA	NA	NA	NA	NA	NA	NA	NA	NA	NA	NA

^1^: Measured in U/L; ^2^: measured in μg/L; * *p* < 0.05; ^+^ *p* < 0.001; NR: not reported; NA: not applicable.

**Table 5:** Postoperative urine Klotho.

Study ID	Postoperative Urine Klotho	
	***AKI***	***Non-AKI***
Torregrosa et al., 2015 [26]	1.97 ± 0.04 *^1^ 1.25 ± 0.33 ^2^	1.80 ± 0.08 1.34 ± 0.36
Yong-Jun Liu et al., 2015 [40]	NR	NR
Yingying Qian et al., 2019 [27]	1.69 ± 2.43 *^3^	0.52 ± 1.18
Ales Jerin et al., 2020 [28]	NR	NR
***Total***	*NA*	*NA*

ID: identification; AKI: acute kidney injury; ^1^: measured in ng/mL at 12 h post-op using the kit by Shanghai Sunred Biological Technology Co., Ltd. (Shanghai, China); ^2^: Measured in ng/mL at 12 h post-op using the kit by IBL International (Human soluble a-Klotho Assay Kit–IBL, Immuno-Biological Laboratories Co., Ltd., Hamburg, Germany); ^3^: measured in ng/umol at the time of transfer to the intensive care unit (<2 h); * *p* < 0.05; NR: not reported; NA: not applicable.

## Supplementary Materials

The following supporting information can be downloaded at: https://www.mdpi.com/article/10.3390/jcdd11050135/s1, Figure S1: PRISMA flow diagram; Table S1: Newcastle-Ottawa rating scale results for the included studies; Table S2: Comorbidities of included patients.

## References

[1] Brown J.K., Shaw A.D., Mythen M.G., Guzzi L., Reddy V.S., Crisafi C., Engelman D.T. (2023). Adult Cardiac Surgery-Associated Acute Kidney Injury: Joint Consensus Report. J. Cardiothorac. Vasc. Anesth..

[2] Vives M., Hernandez A., Parramon F., Estanyol N., Pardina B., Muñoz A., Alvarez P., Hernandez C. (2019). Acute kidney injury after cardiac surgery: Prevalence, impact and management challenges. Int. J. Nephrol. Renov. Dis..

[3] Wang Y., Bellomo R. (2017). Cardiac surgery-associated acute kidney injury: Risk factors, pathophysiology and treatment. Nat. Rev. Nephrol..

[4] Schurle A., Koyner J.L. (2021). CSA-AKI: Incidence, Epidemiology, Clinical Outcomes, and Economic Impact. J. Clin. Med..

[5] Rydén L., Sartipy U., Evans M., Holzmann M.J. (2014). Acute kidney injury after coronary artery bypass grafting and long-term risk of end-stage renal disease. Circulation.

[6] Leacche M., Winkelmayer W.C., Paul S., Lin J., Unic D., Rawn J.D., Cohn L.H., Byrne J.G. (2006). Predicting survival in patients requiring renal replacement therapy after cardiac surgery. Ann. Thorac. Surg..

[7] Alshaikh H.N., Katz N.M., Gani F., Nagarajan N., Canner J.K., Kacker S., Najjar P.A., Higgins R.S., Schneider E.B. (2018). Financial Impact of Acute Kidney Injury After Cardiac Operations in the United States. Ann. Thorac. Surg..

[8] Mehta R.H., Grab J.D., O’Brien S.M., Bridges C.R., Gammie J.S., Haan C.K., Ferguson T.B., Peterson E.D. (2006). Bedside tool for predicting the risk of postoperative dialysis in patients undergoing cardiac surgery. Circulation.

[9] Wijeysundera D.N., Karkouti K., Dupuis J.Y., Rao V., Chan C.T., Granton J.T., Beattie W.S. (2007). Derivation and validation of a simplified predictive index for renal replacement therapy after cardiac surgery. JAMA.

[10] Thakar C.V., Arrigain S., Worley S., Yared J.P., Paganini E.P. (2005). A clinical score to predict acute renal failure after cardiac surgery. J. Am. Soc. Nephrol..

[11] Wiguna I.I., Bhaskara G.N.I., Putri M.R.A., Ardianto P., Atmoko W. (2023). TIMP2 and IGFBP-7 as Biomarkers for The Diagnosis of Acute Kidney Injury (AKI) in Post-operative Patients: An Evidence-Based Case Report. Acta Med. Indones..

[12] Wen Y., Parikh C.R. (2021). Current concepts and advances in biomarkers of acute kidney injury. Crit. Rev. Clin. Lab. Sci..

[13] Kuro O.M. (2019). The Klotho proteins in health and disease. Nat. Rev. Nephrol..

[14] Arking D.E., Krebsova A., Macek M., Macek M., Arking A., Mian I.S., Fried L., Hamosh A., Dey S., McIntosh I. (2002). Association of human aging with a functional variant of klotho. Proc. Natl. Acad. Sci. USA.

[15] Han S., Zhang X., Wang X., Wang Y., Xu Y., Shang L. (2023). Association Between Serum Klotho and All-Cause Mortality in Chronic Kidney Disease: Evidence from a Prospective Cohort Study. Am. J. Nephrol..

[16] Pei Y., Miu M., Mao X., Chen W., Zhu J. (2023). α-Klotho: An Early Risk-Predictive Biomarker for Acute Kidney Injury in Patients with Acute Myocardial Infarction. Int. J. Clin. Pract..

[17] Ni W., Zhang Y., Yin Z. (2021). The protective mechanism of Klotho gene-modified bone marrow mesenchymal stem cells on acute kidney injury induced by rhabdomyolysis. Regen. Ther..

[18] Xu J., Lin E., Hong X., Li L., Gu J., Zhao J., Liu Y. (2023). Klotho-derived peptide KP1 ameliorates SARS-CoV-2-associated acute kidney injury. Front. Pharmacol..

[19] Zhou P., Zhao C., Chen Y., Liu X., Wu C., Hu Z. (2023). Klotho activation of Nrf2 inhibits the ferroptosis signaling pathway to ameliorate sepsis-associated acute kidney injury. Transl. Androl. Urol..

[20] Panesso M.C., Shi M., Cho H.J., Paek J., Ye J., Moe O.W., Hu M.C. (2014). Klotho has dual protective effects on cisplatin-induced acute kidney injury. Kidney Int..

[21] Wang M., Zhou Y., Hao G., Wu Y.E., Yin R., Zheng Y., Zhao W. (2023). Recombinant Klotho alleviates vancomycin-induced acute kidney injury by upregulating anti-oxidative capacity via JAK2/STAT3/GPx3 axis. Toxicology.

[22] Hu M.C., Shi M., Zhang J., Quiñones H., Kuro-o M., Moe O.W. (2010). Klotho deficiency is an early biomarker of renal ischemia-reperfusion injury and its replacement is protective. Kidney Int..

[23] Moreno J.A., Izquierdo M.C., Sanchez-Niño M.D., Suárez-Alvarez B., Lopez-Larrea C., Jakubowski A., Blanco J., Ramirez R., Selgas R., Ruiz-Ortega M. (2011). The inflammatory cytokines TWEAK and TNFα reduce renal klotho expression through NFκB. J. Am. Soc. Nephrol..

[24] Sahu A., Mamiya H., Shinde S.N., Cheikhi A., Winter L.L., Vo N.V., Stolz D., Roginskaya V., Tang W.Y., St Croix C. (2018). Age-related declines in α-Klotho drive progenitor cell mitochondrial dysfunction and impaired muscle regeneration. Nat. Commun..

[25] Donate-Correa J., Martín-Carro B., Cannata-Andía J.B., Mora-Fernández C., Navarro-González J.F. (2023). Klotho, Oxidative Stress, and Mitochondrial Damage in Kidney Disease. Antioxidants.

[26] Torregrosa I., Montoliu C., Urios A., Giménez-Garzó C., Tomás P., Solís M., Ramos C., Juan I., Puchades M.J., Saez G. (2015). Urinary Klotho measured by ELISA as an early biomarker of acute kidney injury in patients after cardiac surgery or coronary angiography. Nefrologia.

[27] Qian Y., Che L., Yan Y., Lu R., Zhu M., Xue S., Ni Z., Gu L. (2019). Urine klotho is a potential early biomarker for acute kidney injury and associated with poor renal outcome after cardiac surgery. BMC Nephrol..

[28] Jerin A., Mosa O.F., Kališnik J.M., Žibert J., Skitek M. (2020). Serum Klotho as a marker for early diagnosis of acute kidney injury after cardiac surgery. J. Med. Biochem..

[29] Kuro-o M., Matsumura Y., Aizawa H., Kawaguchi H., Suga T., Utsugi T., Ohyama Y., Kurabayashi M., Kaname T., Kume E. (1997). Mutation of the mouse klotho gene leads to a syndrome resembling ageing. Nature.

[30] Mylonas K.S., Sarantis P., Kapelouzou A., Karamouzis M.V., Kapetanakis E.I., Kontzoglou K., Iliopoulos D.C., Nikiteas N., Schizas D. (2022). Mechanosensitive Stem-Cell Genes and Klotho in Atherosclerotic Aortas: Regulating Spatially Deranged Expression Patterns Using Colchicine Regimens. J. Clin. Med..

[31] Mylonas K.S., Peroulis M., Kapetanakis E.I., Kapelouzou A. (2024). Myocardial Expression of Pluripotency, Longevity, and Proinflammatory Genes in the Context of Hypercholesterolemia and Statin Treatment. J. Clin. Med..

[32] Hu M.C., Kuro-o M., Moe O.W. (2013). Renal and extrarenal actions of Klotho. Semin. Nephrol..

[33] Medici D., Razzaque M.S., Deluca S., Rector T.L., Hou B., Kang K., Goetz R., Mohammadi M., Kuro O.M., Olsen B.R. (2008). FGF-23-Klotho signaling stimulates proliferation and prevents vitamin D-induced apoptosis. J. Cell Biol..

[34] van Goor M.K.C., Hoenderop J.G.J., van der Wijst J. (2017). TRP channels in calcium homeostasis: From hormonal control to structure-function relationship of TRPV5 and TRPV6. Biochim. Biophys. Acta Mol. Cell Res..

[35] Imura A., Tsuji Y., Murata M., Maeda R., Kubota K., Iwano A., Obuse C., Togashi K., Tominaga M., Kita N. (2007). α-Klotho as a regulator of calcium homeostasis. Science.

[36] Page M.J., McKenzie J.E., Bossuyt P.M., Boutron I., Hoffmann T.C., Mulrow C.D., Shamseer L., Tetzlaff J.M., Akl E.A., Brennan S.E. (2021). The PRISMA 2020 statement: An updated guideline for reporting systematic reviews. J. Clin. Epidemiol..

[37] Hozo S.P., Djulbegovic B., Hozo I. (2005). Estimating the mean and variance from the median, range, and the size of a sample. BMC Med. Res. Methodol..

[38] Higgins J.P.T., Thomas J., Chandler J., Cumpston M., Li T., Page M.J., Welch V.A. (2023). Cochrane Handbook for Systematic Reviews of Interventions Version 6.4.

[39] Lo C.K., Mertz D., Loeb M. (2014). Newcastle-Ottawa Scale: Comparing reviewers’ to authors’ assessments. BMC Med. Res. Methodol..

[40] Liu Y.J., Sun H.D., Chen J., Chen M.Y., Ouyang B., Guan X.D. (2015). Klotho: A novel and early biomarker of acute kidney injury after cardiac valve replacement surgery in adults. Int. J. Clin. Exp. Med..

[41] Samra M., Abcar A.C. (2012). False estimates of elevated creatinine. Perm. J..

[42] Gwinner W., Hinzmann K., Erdbruegger U., Scheffner I., Broecker V., Vaske B., Kreipe H., Haller H., Schwarz A., Mengel M. (2008). Acute tubular injury in protocol biopsies of renal grafts: Prevalence, associated factors and effect on long-term function. Am. J. Transplant..

[43] Moledina D.G., Hall I.E., Thiessen-Philbrook H., Reese P.P., Weng F.L., Schröppel B., Doshi M.D., Wilson F.P., Coca S.G., Parikh C.R. (2017). Performance of Serum Creatinine and Kidney Injury Biomarkers for Diagnosing Histologic Acute Tubular Injury. Am. J. Kidney Dis..

[44] Shi M., Flores B., Gillings N., Bian A., Cho H.J., Yan S., Liu Y., Levine B., Moe O.W., Hu M.C. (2016). αKlotho Mitigates Progression of AKI to CKD through Activation of Autophagy. J. Am. Soc. Nephrol..

[45] Adema A.Y., Vervloet M.G., Blankenstein M.A., Heijboer A.C. (2015). α-Klotho is unstable in human urine. Kidney Int..

[46] Qian Y., Guo X., Che L., Guan X., Wu B., Lu R., Zhu M., Pang H., Yan Y., Ni Z. (2018). Klotho Reduces Necroptosis by Targeting Oxidative Stress Involved in Renal Ischemic-Reperfusion Injury. Cell. Physiol. Biochem..

[47] Pode Shakked N., Rosenblat O., Sagiv D., Molad J., Weinberg H., Shlomo M., Tokatly Latzer I., Pleniceanu O., Mishali D., Vardi A. (2021). Protective effect of soluble Klotho in pediatric patients undergoing cardiac surgery with cardiopulmonary bypass support-A pilot study. J. Card. Surg..

[48] Fu Y., Cao J., Wei X., Ge Y., Su Z., Yu D. (2023). Klotho alleviates contrast-induced acute kidney injury by suppressing oxidative stress, inflammation, and NF-KappaB/NLRP3-mediated pyroptosis. Int. Immunopharmacol..

