# Supplementary material for: Klotho as an Early Marker of Acute Kidney Injury Following Cardiac Surgery: A Systematic Review
Source: J Cardiovasc Dev Dis. 2024 Apr 28;11(5):135. doi: 10.3390/jcdd11050135 (PMC11122318; doi:10.3390/jcdd11050135)
Supplement: Supplementary file 1 [file jcdd-11-00135-s001.zip › jcdd-2910103-supplementary.pdf]

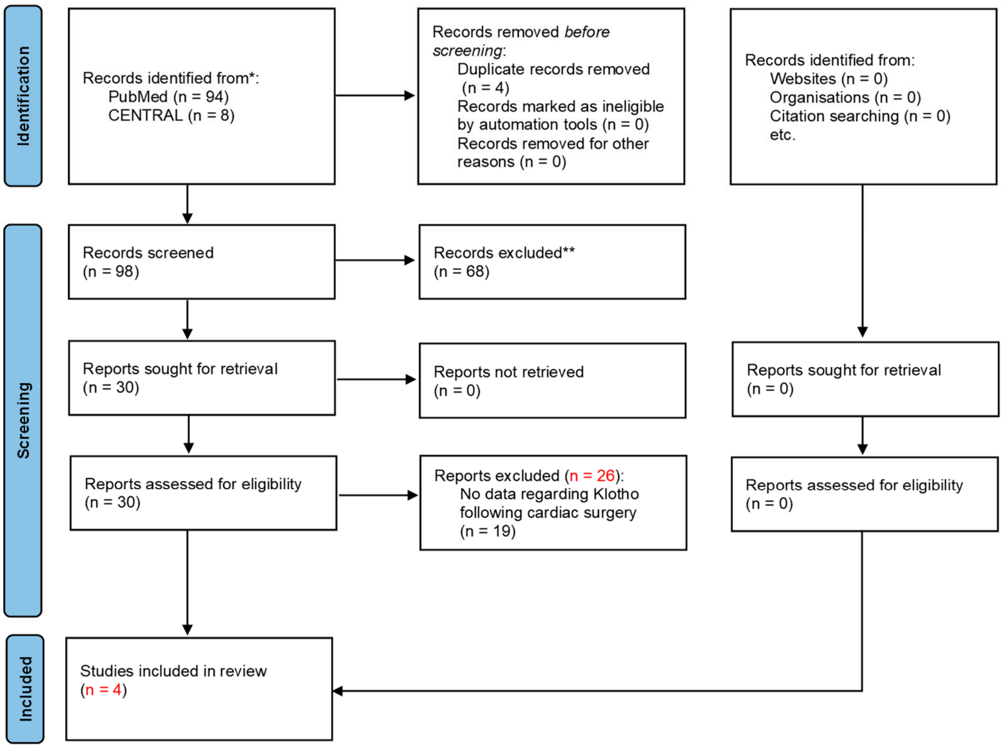

For more information, visit: http://www.prisma-statement.org/

Supplemental Figure S1. PRISMA flow diagram [36]

Supplemental Table S1. Newcastle-Ottawa rating scale results for the included studies

Study ID	Representativeness	Selection of non-exposed	Ascertainment of exposure	Outcome not present at start	Comparability on major factor	Comparability on other factors	Assessment of outcome	Long enough follow-up	Adequacy of follow-up	Total score
**Torregrosa 2015［26］**	1	1	1	1	1	0	1	1	1	8
**Yong-Jun Liu 2015 [40]**	1	1	1	1	1	0	1	1	1	8
**Yingying 2019 [27]**	1	1	1	1	1	0	1	1	1	8
**Ales Jerin 2020 [28]**	1	1	1	1	1	0	1	1	1	8

Supplemental Table S2. Comorbidities of included patients

**Study ID**	**Hypertension**		**Dyslipidemia**		**Congestive heart failure**		**Peripheral artery disease**		**Cerebrovascular disease**		**Diabetes mellitus**	
	AKI	***Non-AKI***	***AKI***	***Non-AKI***	***AKI***	***Non-AKI***	***AKI***	***Non-AKI***	***AKI***	***Non-AKI***	***AKI***	***Non-AKI***
Torregrosa et al., 2014 [26]	NR	NR	NR	NR	NR	NR	NR	NR	NR	NR	NR	NR
Yong-Jun Liu et al., 2015 [40]	4/19 (21.1%)	2/16 (12.5%)	NR	NR	NR	NR	NR	NR	NR	NR	3/19 (15.7%)	1/16 (6.2%)
Yingying Qian et al., 2019 [27]	13/33 (39.4%)	20/58 (34.5%)	1/33 (3.0%)	5/58 (8.6%)	7/33 (21.2%)	6/58 (10.3%)	NR	NR	0/33 (0%)	4/58 (6.9%)	6/33 (18.1%)	8/58 (13.8%)

Ales Jerin et al., 2020 [28]	10/52 (19.2%)	6/26 (23.1%)	NR	NR	NR	NR	NR	NR	NR	NR	16/52 (30.7%)	9/26 (34.6%)
Total	*27/104 (25.9%)*	*28/100 (28%)*	*1/33 (3%)*	*5/58 (8.6%)*	*7/33 (21.2%)*	*6/58 (10.3%)*	*NR*	*NR*	*0/33 (0%)*	*4/58 (6.9%)*	*25/104 (24%)*	*18/100 (18%)*

ID: identification; AKI: acute kidney injury; NR: not reported
